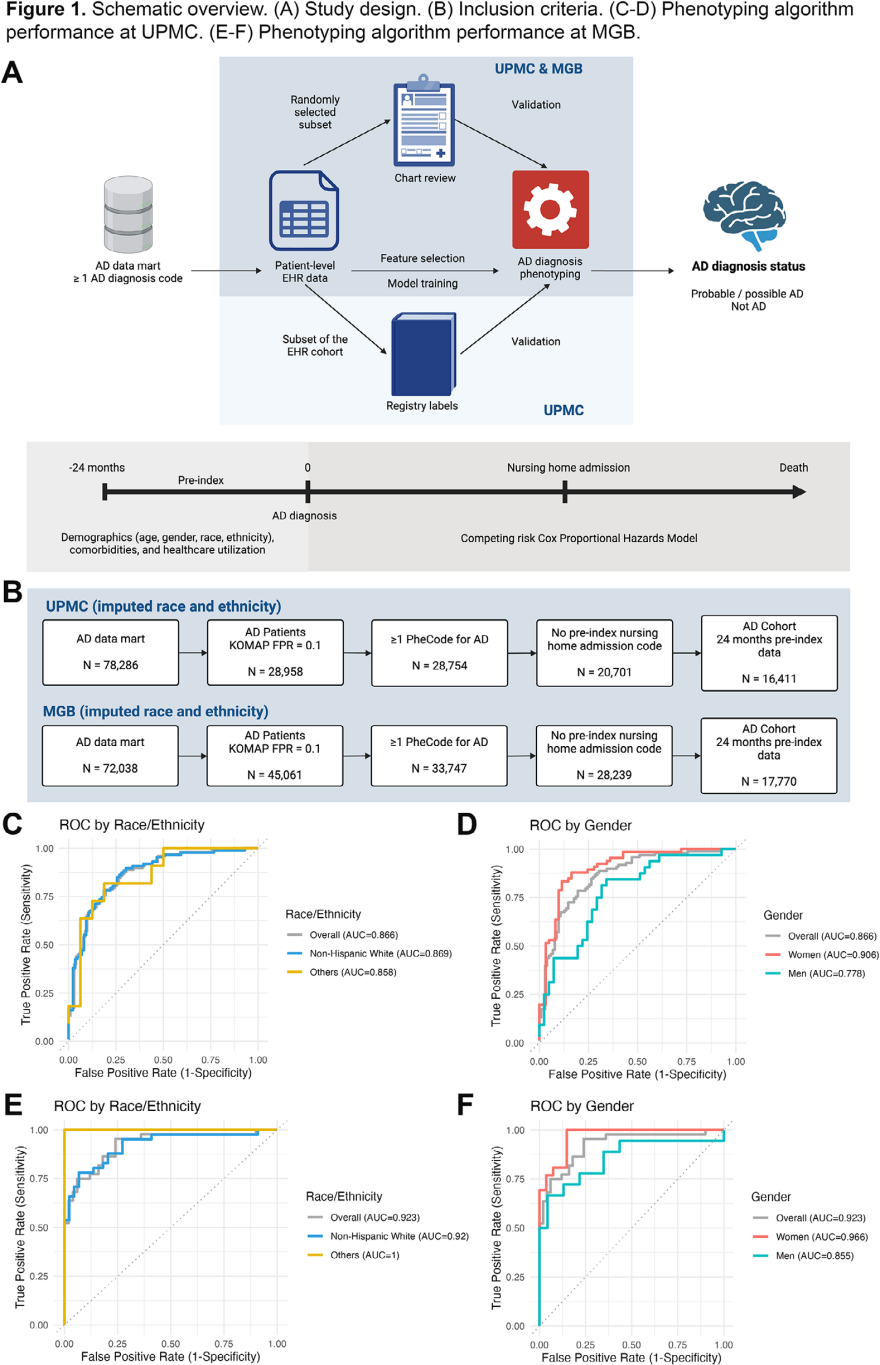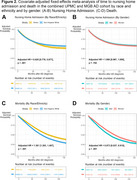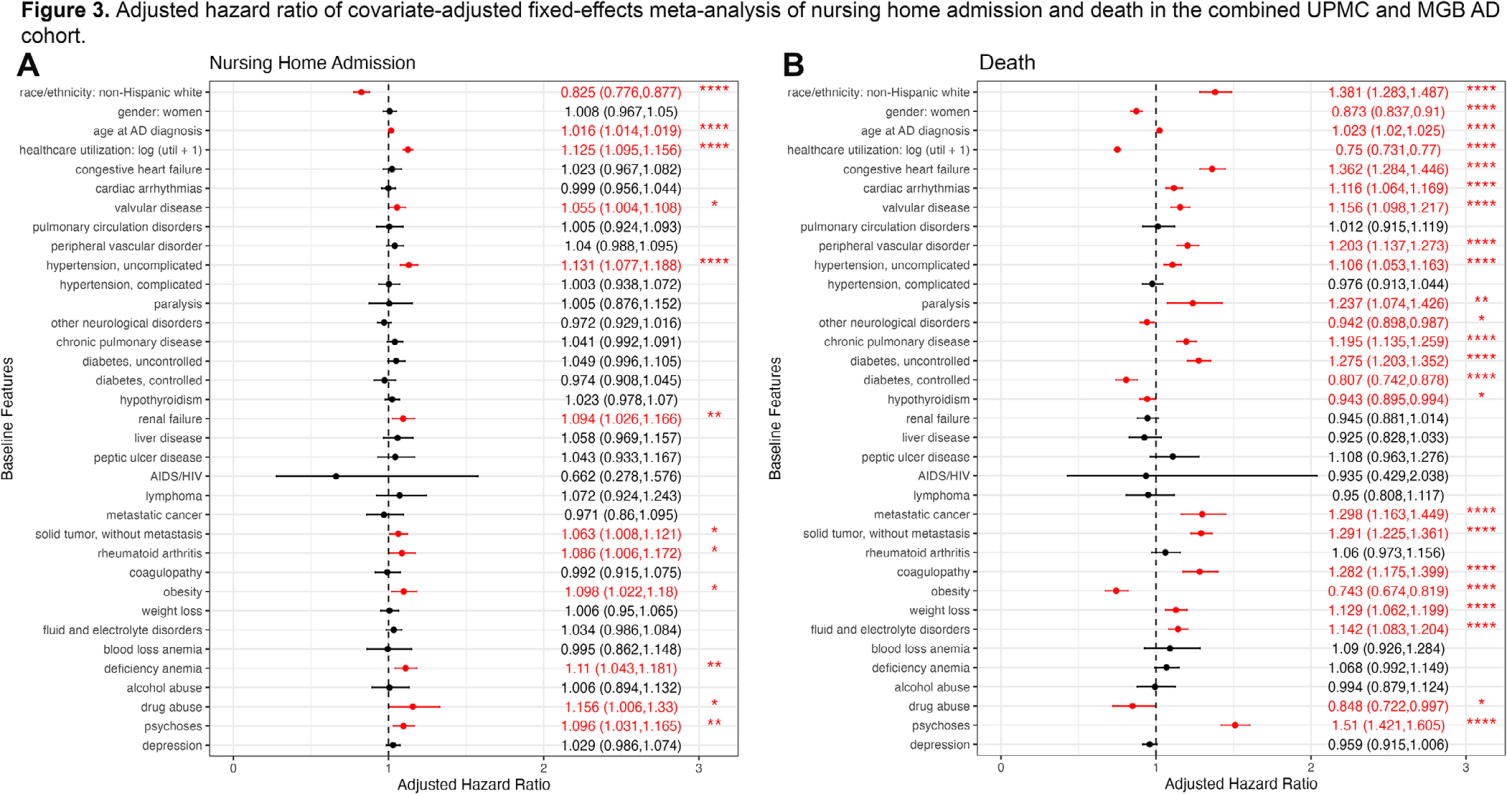# Leveraging electronic health records to examine outcome disparities in people with Alzheimer's Disease

**DOI:** 10.1002/alz70860_099807

**Published:** 2025-12-23

**Authors:** Shruthi Venkatesh, Linshanshan Wang, Michele Morris, Mohammed Moro, Ratnam Srivastava, Yunqing Han, Riddhi Patira, Sarah B Berman, Oscar L Lopez, Shyam Visweswaran, Tianrun Cai, Tianxi Cai, Zongqi Xia

**Affiliations:** ^1^ University of Pittsburgh, Pittsburgh, PA, USA; ^2^ Harvard T.H. Chan School of Public Health, Boston, MA, USA; ^3^ Harvard Medical School, Boston, MA, USA; ^4^ Brigham and Women's Hospital, Boston, MA, USA

## Abstract

**Background:**

Alzheimer's disease (AD) carries a high societal burden that is inequitably distributed across demographic groups. The objective of this study is to examine differences in readily ascertainable outcomes of AD decline by race and ethnicity and by gender.

**Method:**

Using electronic health record (EHR) data from two large healthcare systems spanning 1994 to 2022, we identified patients with at least one diagnosis code for AD or related dementia. We applied an unsupervised phenotyping algorithm at each site to predict AD diagnosis status, which was validated with gold‐standard chart‐reviewed and registry‐derived diagnosis labels. After excluding patients with less than 24 months of data or who were admitted to nursing homes prior to AD diagnosis, we examined the time to two outcomes of AD decline, nursing home admission and death, in survival analyses stratified by demographic groups. We accounted for baseline covariates (age, gender, race, ethnicity, healthcare utilization, and comorbidities) in the survival analysis. We then performed a fixed‐effects meta‐analysis of the survival data from both healthcare systems.

**Result:**

The phenotyping algorithm demonstrated high accuracy in identifying AD patients across both healthcare systems (AUROC score range: 0.835‐0.923). Of the 34,181 AD patients included (62% women, 90% non‐Hispanic White, 80.39±9.28 years of age at AD diagnosis), 32% were admitted to nursing homes and 50% died. In the fixed‐effect meta‐analysis, non‐Hispanic White patients had a lower risk of nursing home admission (HR[95% CI]=0.825[0.776‐0.877], *p* <0.001) and higher risk of death (HR[95% CI]=1.381[1.283‐1.487], *p* < .0001) than racial and ethnic minorities. There was no difference between women and men in the risk of nursing home admission (HR[95% CI]=1.008[0.967‐1.050], *p* = .762), but women had a lower risk of death (HR[95% CI]=0.873[0.837‐0.910], *p* < .0001) than men.

**Conclusion:**

Racial and ethnic minorities with AD may have a higher risk of nursing home admission, whereas non‐Hispanic White patients and men with AD may have a higher risk of death. Future investigation of demographic disparity in AD decline can inform clinical management and public health policies.